# CENPA a Genomic Marker for Centromere Activity and Human Diseases

**DOI:** 10.2174/138920209788920985

**Published:** 2009-08

**Authors:** Manuel M. Valdivia, Khaoula Hamdouch, Manuela Ortiz, Antonio Astola

**Affiliations:** Departamento de Bioquímica y Biología Molecular, Facultad de Ciencias, Universidad de Cádiz, 11510 Puerto Real, Cádiz, Spain

**Keywords:** CENPA, centromere, kinetochore, histone H3-like variant, alphoid DNA, epigenetic, autoantigen, scleroderma, aneuploidy, cancer.

## Abstract

Inheritance of genetic material requires that chromosomes segregate faithfully during cell division. Failure in this process can drive to aneuploidy phenomenon. Kinetochores are unique centromere macromolecular protein structures that attach chromosomes to the spindle for a proper movement and segregation. A unique type of nucleosomes of centromeric chromatin provides the base for kinetochore formation. A specific histone H3 variant, CENPA, replaces conventional histone H3 and together with centromere-specific-DNA-binding factors directs the assembly of active kinetochores. Recent studies on CENPA nucleosomal structure, epigenetic inheritance of centromeric chromatin and transcription of pericentric heterochromatin provide new clues to our understanding of centromere structure and function. This review highlights the role and dynamics of CENPA assembly into centromeres and the potential contribution of this kinetochore protein to autoimmune and cancer diseases in humans.

## INTRODUCTION

Every eukaryotic chromosome requires a proteinaceous structure, the kinetochore that forms the interphase between centromeric DNA and the microtubules that pull the chromosomes to the poles at mitosis and meiosis. A complex group of proteins builds the kinetochore from the DNA up [1]. The foundation of the kinetochore is the centromere-specific nucleosomes, where a histone H3-like variant, CENPA, replaces ordinary histone H3 in centromeric chromatin. The essential cellular function played by CENPA in determining kinetochore assembly, centromere activity, chromosome stability and cell viability is well established. Failure in any of those events is expected to be linked in some way to cancer, infertility, mental disorders, ageing and degenerative diseases.

Current centromere models indicate that, once formed, centromeres are specified epigenetically and maintained at the same locus cell division after cell division. CENPA has emerged as the best candidate to carry the epigenetic centromere mark whereby it confers conformational rigidity to the nucleosome [2]. Recent reviews on centromere organization and epigenesis and kinetochore-microtubule dynamics have been published [3-7]. In this report we focus on the structure and function of CENPA in kinetochore assembly and centromere activity, and we detail the role of this histone variant in the developing of some human diseases such as cancer and autoimmunity.

### CENPA a Histone H3-Like Variant of Centromeric Nucleosomes

CENPA is an 18-kDa protein firstly identified in humans by autoantibodies found in patients suffering scleroderma [8]. Initial immunofluorescence analysis served to show that CENPA is concentrated at the kinetochore in centromere regions of metaphase chromosomes [9, 10] Fig. (**1**). Localization of CENPA by electron microscopy has shown that it occupies a compact domain at the inner kinetochore plate [11]. The kinetochore-forming CENPA chromatin exists as disk shape of 0.5-1 µm in diameter tight to the centromere that is though to exclude H3-containing nucleosomes [12]. In an average human centromere, 15000 copies of the CENPA-containing nucleosomes are estimate to lead to a layer(s) of CENPA chromatin at the foundation of the kinetochore [13]. The higher-order organization of CENPA nucleosomes is though to be not contiguous on physically stretched chromatin fibers but is brought together in three dimensional spaces to a multisubunit domain [14]. At the biochemical level, CENPA is a centromere-specific homolog of the core nucleosomal protein histone H3 [15]. CENPA replaces histone H3 at the centromere nucleosomes where it has unique structural properties essential for centromere functions [16]. CENPA-associated mononucleosomes have a molecular mass of ~200 kDa and comprise 120–150 bp of DNA and equimolar amounts of CENPA and histones H4, H2A and H2B, forming an octameric complex [17-19]. However, unlike the core histones, CENPA is retained in bovine sperm nuclei in discrete foci [20].

By chromatin immunoprecipitation (ChIP), the CENPA-associated human DNA was identified as the alpha-satellite (alphoid) DNA, the major constituent of human centromeres [21]. This DNA is organized in tandem repeated arrays of a 171 base pairs in which a 17bp motif, named the CENPB box, is the binding site for the centromere protein CENPB [21]. This binding promotes nucleosome phasing between CENPB boxes. CENPA-containing nucleosomes are phased on alphoid DNA as a result of interactions between CENPB and CENPB boxes. Further, the presence of CENPB boxes had a strong impact on the nucleation of functional kinetochores in human artificial chromosomes (HACs), which support a multisubunit repetitive model for the organization of mammalian centromeres [14, 22]. While CENPB is located extensively along human α–satellite DNA repeats at the innermost heterochomatic regions of the centromeres, CENPA only bind along a small portion of the alphoid DNA located outside of CENPB at the inner plate of the kinetochore [11].

CENPA nucleosomes are interspersed in the centromere with nucleosomes containing histone H3 dimethylated at Lys4, thus distinguishing centromeric chromatin (CEN chromatin) from flanking heterochromatin, which is defined by H3 methylation at Lys9 [19]. CENPA and histone H4 form subnucleosomal tetramers that are more compact and conformationally more rigid than the corresponding tetramers of histones H3 and H4. Recently, studies of cross-linked nucleosomes in *Drosophila melanogaster *interphase cells, served to detect heterotypic tetramers containing one copy each of CenH3 (CID), H2A, H2B, and H4 [16]. Atomic force microscopy reveals that native CenH3 containing nucleosomes are only half as high as canonical octameric nucleosomes [16]. This result of stable half tetrameric structure of nucleosomes *in vivo* provides new evidences to pursue for understanding centromeric chromatin structure and identity. In yeast, cohesion at the pericentromeric regions affect kinetochore geometry in meiosis I but this cohesion is prevented during mitosis. Therefore some mechanisms required for establishing centromere cohesion might be intrinsically suppressed in the CENPA nucleosome region [23].

### Structural Features of CENPA Protein

CENPA homologous proteins have been studied and characterized in many species, such as budding yeast (*Cse4*) [24], fission yeast (*Cnp1*) [25], *Candida albicans* [26], fly (*CID*) [27], nematodes [28], plants [29], amphibians [30], birds [31] and other mammals such as bovine, hamster, mice and rat in addition to human [32, 33]. Experiments in budding yeast demonstrated that CENPA can structurally and functionally be substitute for human CENPA, strongly suggesting that the basic features of this protein are conserved between yeasts and mammals [34].

Human CENPA has 140 amino acids residues with a 51% sequence identical to that of regular histone H3. All known CENPA proteins from yeast to human have a unique NH_2_-terminal domain that differs completely from that of histone H3, and that appears to be important for interaction with other kinetochore components, Fig. (**2**) [35-37]. In contrast, the COOH-terminal domain shares 62% identity with the nucleosomal core histone H3 [38]. A short domain was identified in the C-terminal region of CENPA that is necessary for efficient targeting to centromeres Fig. (**2**) [19]. The so-called CENPA targeting domain (CATD) was identified in yeast and humans to consist of both loop 1 and α2 helix on the CENPA structure, Fig. (**2**) [39, 40]. Mammals CENPA differs from histone H3 within the CATD domain by a similar number of amino acid insertions and substitutions (22 out of 42 residues) as the yeast CENPA homolog, Cse4p (18 out of 43 residues) [13].

Although the CENPA nucleosomes have been successfully reconstituted [41,42], the protein has far resisted crystallographic approaches. Attempts to express the CENPA gene in *E.coli* were initially unsuccessful probably because the human gene contains 28 minor codons for *E.coli* [41, 43]. However a new gene optimized for expression in *E. coli *was constructed, and purified recombinant CENPA formed a nucleosome-like structure with histones H2A, H2B and H4 [41]. A deuterium exchange/mass spectrometry-based strategy has been used to structurally characterized CENPA nucleosomes [44]. It was found a striking structural difference in rigidity between CENPA and histone H3 nucleosomes mediated by the short CENPA targeting domain CATD in the histone fold [44]. The findings indicated rigid physical properties of the nucleosomes harboring CENPA/H4 tetramers that were independent of DNA sequence [2]. Further, it was described that the centromere identity can be maintained in nucleosomes where protein chimaeras in which the CATD domain from CENPA is swapped into histone H3 target to centromeres and maintain cell viability when endogenous CENPA levels are significantly reduced [7, 44].

### CENPA is Essential for Centromere Activity and Cell Viability

Knockdown of the CENPA gene is lethal in the early embryonic stages of mouse development [45]. Heterozygous mice were healthy and fertile but null mutants fail to survive beyond 6.5 days post conception [46]. Affected embryos showed severe mitotic problems including micronuclei and macronuclei formation, chromatin fragmentation and hyper condensation. Immunofluorescence analysis of interphase cells at day 5.5 reveals complete CENPA depletion and dispersion of CENPB and CENPC (other kinetochore components) throughout the nucleus. The findings demonstrated that CENPA is essential for kinetochore assembly and organizing centromeric chromatin [45, 46]. Similarly, in the yeast *Saccharomyces cerevisiae*, chromosome misssegregation mutant *cse4-1*, the homolog of human CENPA, has been isolated and shown to increase the no disjunction frequency of a chromosome bearing a mutant centromere DNA sequence [47]. The *Candida albicans* CENPA homolog (*CaCse4p*) is essential for cell viability of diploid cells where cells depleted of CENPA, possessed a mitosis-specific arrest phenotype, with accumulation of large-budded cells containing single G_2_ nuclei [48]. Induced chromosome instability and missegregation were also found in D-40 chicken cells that were null for the CENPA gene [49]. In *Drosophila*, injection of antibodies against CID (the fly protein homologous to human CENPA) into early embryos, as well as RNAi studies in cultured cells, showed that CID is required for several mitotic processes [50].

RNAi experiments have also demonstrated the essential role of CENPA in kinetochore assembly. Localization of the constitutive inner kinetochore protein complex (NAC-CAD see below), is abolished in CENPA-RNAi cells [51]. The CENPA nucleosome associated complex (NAC) is essential to load CAD components at the kinetochore and disruption of the complex causes errors of chromosome alignment and segregation. This is observed when the CENPA gene is annulled in HeLa [51] and *C. elegans* [52]. However, recruitment of normal levels of kinetochore proteins, centromere-generated mitotic checkpoint signalling, chromosome segregation and viability can be rescue by histone H3 carrying the CENPA targeting domain CATD [13, 36]. Based on these observations, CENPA is observed as critical factor for the assembly of a macromolecular protein complex at the kinetochore for centromere activity.

### CENPA Assembly at the Kinetochore and Association with other Centromere Components

The centromere contains constitutive proteins permanently associated during the cell cycle, as well as other proteins just transiently bound and known as “passenger centromere proteins”. Examples of constitutive centromere proteins are the human autoantigens CENPA, CENPB and CENPC than bound to the I-type α satellite array constitutes the pre-kinetochore in human cells [9, 53]. A number of CENPA-associated proteins have been identified in human cells by methods based on ChIP and mass spectroscopy [54, 55]. CENPA-containing nucleosomes directly recruit a proximal protein complex (NAC) comprising of six components including CENPC, CENPM, CENPN, CENPT, CENPU and CENPH that together with CENPI form the inner kinetochore plate. The CENPA-NAC complex serves to load a more distal inner kinetochore protein complex of CENPK, CENPL, CENPO, CENPQ, CENPR and CENPS (named the CAD complex) that do not recognize CENPA nucleosomes directly [55, 56]. The CENPA-NAC-CAD complexes are referring as the constitutive centromere-associated network (CCAN) and are only partially conserved in all eukaryotes [7].

Although CENPA nucleosomes is the base to constitute a nucleation site for kinetochore assembly, mutual interactions and dependency between some of its components drive the organization of the CENPA-NAC complex, Fig. (**3**). Depletion of CENPA by shRNA have no effects on the localization of the alphoid binding sequence CENPB but eliminated CENPH, CENPM and CEPN [55]. Localization of all components of the NAC-CAD complexes are abolished in CENPA RNAi cells [51]. Also RNAi experiments demonstrated that CENPA, CENPH and CENPI are all required to recruit CENPC to the kinetochore [57,58]. At the same time, the CENPH/I complex may function in part for targeting of newly synthesized CENPA to centromeres (see below) [54, 59].

Biochemical and bioinformatics studies have demonstrated that kinetochores contain another structural protein core called KMN, constituted at least by three complexes, Mis12, Ndc 80 and KNL1. The KMN network is conserved in all eukaryotes, directly binds to microtubules and co-ordinately with the CENPH/I complex direct the kinetochore assembly in vertebrates [7, 60, 61]. Initial findings by RNAi experiments indicated that kinetochore localization dependency does not appear to exist between CENPA and human Mis12 (hMis12) [51]. Although CENPA localization was unaffected, centromeric signals for CENPH and CENPI were decreased upon depletion of hMis12 [51, 62]. Further, human Mis12 can be found at the centromere in spite of the removal of CENPA, CENPI and CENPC [51]. Therefore siRNA experiments in human cells, revealed that Mis12 and CENPA could act independently to recruit CENPI and CENPH and localization of CENPA and hMis12 are independent [51,58]. More recently, in yeast and human cells, Mis12 was found to work in a complex with Nnf1, Nsl1, and Dsn1 [61]. The four subunit complexes co-localize with human CENPA at inner kinetochore but only in a subset of interphase cells, suggesting that the hMis12 complex is not constitutively present at centromeres throughout the cell cycle [61]. RNAi–mediated depletion of the hMis12 complex indicated that localization of the four subunit is interdependent [61, 62] and revealed a reduction in CENPA levels in cells depleted of the complex [61]. At the same time, localization of hMis12 was clearly but not completely affected by RNAi-mediated depletion of CENPA [62]. These results could be explained if different pools of hMis12 complex exist whose localization at centromeres is partially independent of CENPA [61, 62].

A new kinetochore factor, KNL2, was identified to be conserved from nematodes to human. This protein although is only transiently present at centromeres after mitotic exit, was found to be required specifically for CENPA loading and kinetochore assembly [63]. Recently a new centromere component CENPW was found to forms a complex with CENPT that is directly associated with nucleosomal DNA and with canonical histone H3 but not CENPA in centromeric regions. The CENPT/CENPW complex functions upstream of other NAC components and is directly involved in establishment of centromere chromatin structure coordinately with CENP-A [64].

Among the group of passenger proteins transiently associated with the centromere, some of them interact with CENPA and govern the progress through mitosis. Thus, Aurora-B, concentrates at centromeres in early G_2_ phase at the time that histone H3 and CENPA are phosphorylated. This kinase phosphorylates H3 and CENPA at an equivalent target serine residue [65]. A two-hybrid screen served also to identify CENPA as a protein that interacts with STK6 (Aurora-A) [66]. This kinase phosphorylated CENPA *in vitro* on Ser7, a residue also known to be targeted by Aurora- B. Even more, Aurora-C a kinase only been found in mammals, share with Aurora-B a role in phosphorylation and regulation of CENPA during mitosis [67]. CENPA phosphorylation may be part of the epìgenetic mechanism for kinetochore assembly and preventing it led to chromosome misalignment during mitosis. Several protein interactions may also contribute to the role of CENPA in kinetochore assembly and function. Thus, CENPA also interacts with PARP1 (Poly [ADP-ribose] polymerase 1) driving to a significant poly-ADP-ribosylation upon gamma-irradiation of cells [68]. In budding yeast, two-hybrid studies showed that yeast Scm3, a non-histone protein, and yeast CENPA interact *in vivo*, and chromatin immunoprecipitation assays revealed that Scm3, like yeast CENPA, is found associated with centromeric DNA [69, 70, 71]. The levels of budding yeast CENPA are also regulated by ubiquitin–proteasome-mediated proteolysis, which constitutes a mechanism contributing to the restricted centromere localization of this yeast centromeric histone H3 variant [72]. Finally, the histone chaperone and remodelling complex FACT, a RNA polymerase II cofactor, interacts with centromeric CENPA nucleosomes and is required for centromere-heterochromatin integrity and accurate chromosome segregation [73].

### CENPA is Part of an Epigenetic Mechanism for Centromere Organization and Activity

Although CENPA proteins have been conserved during evolution, paradoxically, centromere DNA sequences are highly variable among species [74]. Remarkably, heritable states at the centromere can be propagated independently of the underlying centromeric DNA sequences. Therefore, the molecular recognition events necessary for kinetochore assembly at the centromere take place at the level of DNA conformation or epigenetic mechanisms rather than DNA sequence per se [6]. A candidate for an epigenetic mark of kinetochore is the specific chromatin factor at the centromere CENPA. As a constitutive centromere protein, CENPA is present during the entire cell cycle, and it should be recruited to the centromere during replication or postreplication in order to supply new CENPA-containing nucleosomes to the duplicated sister centromeres. Examples from low eukaryotes and plants support this notion because CENPA homologues appear to assemble at centromeres before the entry of mitosis [75, 76]. However, in animal cells chromosome segregation proceeds having centromeres filled only with half of the available sites for CENPA [77]. CENPA already bound to mature centromere is quantitatively and equally partitioned to sister centromeres generated during S phase (replication) thereby remaining stably associated through multiple cell divisions. However although centromere DNA replicates in S-phase, CENPA is not expressed until G2 [19] and loading of newly synthesized CENPA is retarded until telophase and the subsequent G1 phase [77]. This indicates that CENPA chromatin assembly occurs through a mechanism distinct from replication-coupled chromatin assembly such as that observed for other histone variants. These steps of temporal assembly of CENPA at centromeric nucleosomes will require passage through an entire cell cycle for proper mitotic function. Incorporation of newly synthesized CENPA occurs without dynamic exchange of already loaded molecules as happen for other CENP molecules such as CENPB, CENPC CENPH and Mis12 [78]. This phenomenon is part of the mechanism for centromere identity and epigenesis.

Activities that transiently associate with kinetochores until anaphase could serve to mark the sites of assembly of newly synthesized CENPA. Thus, the human Mis18 α/β complex, RbAp46/48 and M18BP1 are located at the centromere in a cell-cycle-dependent manner [79, 80]. They accumulate from late anaphase-telophase to early G1 being diffuse throughout the nucleus during other cell cycles steps. The human Mis18 α/β complex and M18BP1 may prime or license the telophase and G1 centromere for the later incorporation of the novo-synthesized CENPA. Both factors could function together with the chaperone RbAp46/48 to generate a chromatin environment permissive for CENPA incorporation. This priming event appears to involve transient histone H4 acetylation at the centromere [79, 80]. Thus, although these factors may not interact directly with CENPA nucleosomes the complexes are regarded as upstream factors for CENPA loading in telophase-G1. In human, marking the centromere for CENPA incorporation in early G1 may require, in addition to the transiently binding loading factors Mis18 complex and M18BP1, stably associated “platform” proteins such as CENPI and CENPH [54].

In the fission yeast *Schizosaccharomyces pombe*, additionally to the Mis18 complex, two factors, Ams2 and Mis6, are required for the correct centromere localization of CENPA (Cnp1). Depletion of Ams2, a cell-cycle-regulated transcription factor, results in the reduction of CENPA binding to the centromere and chromosome missegregation [81, 82]. By contrast, Mis6 is probably required for recruiting fission yeast CENPA to aid forming correct connections between sister centromeres [25]. We like to summarize that CENPA loading at the centromere during the cell cycle may be a common feature but it is certainly not universal because alternative loading pathways were suggested for fission yeast [83], Arabidopsis [76], and Drosophila [84].

In humans, evidences that centromeric DNA is not necessary for kinetochore formation are the fact that non-centromeric DNA in humans (neocentromeres) can acquire and faithfully propagate centromere proteins and functions without any change in the DNA sequence [85]. By genomic array hybridization, neocentromeres have been shown to contain no centromeric alpha-satellite, classical satellites or other known pericentric repetitive sequence motifs but do include a higher AT content similar to that seen in human alpha-satellite DNA [86]. However, CENPA is found on all known, naturally occurring neocentromeres lacking alpha-satellite repeats [87] but it is absent from an inactivated centromere in a dicentric chromosome [88]. The correspondence with active, but not inactive centromeres, supports the notion that CENPA-containing centromeric chromatin might specify the heritable epigenetic nature of the centromere [77].

Transcription of centromere DNA repeat sequences is a novel event that must be considered to understand the complex epigenetic mechanisms that underlie maintenance of centromere structure and function [89]. In eukaryotic chromosomes, heterochromatin is frequently found near CENPA chromatin at the centromere. This heterochromatin is known to contribute to centromere function by ensuring physical cohesion between sisters chromatids [90]. Recent studies have shown that repeated elements embedded within heterochromatic domains are transcribed in order to participate in a mechanism of heterochromatin assembly and silencing [91]. Thus, heterochromatin assembly (including that of the centromere) is believed to occur by an RNAi-mediated mechanism [92]. In human cells, loss of the endoribonuclease DICER, a component of the RNAi machinery, results in cell death accompanied by the accumulation of abnormal mitotic cells that show premature separation of sister chromatids [93]. In DICER-deficient cells, transcripts from human centromeric alpha-satellite repeated sequences accumulate. In those cells, the localization of core kinetochore proteins such as the centromere protein CENPA was normal [93]. Similarly, centromere-based transcription events have been demonstrated in genomes as diverse as those of yeasts [94], maize and rice [95, 96], *Arabidopsis* [97], mouse [98] and chicken [93]. In the fission yeast centromere, the RNA interference-directed heterochromatin flanking the central kinetochore domain is required to promote CENPA (Cnp1) over a central domain of centromeric DNA [99]. Thus, specific histone modifications and RNAi-related progress contribute to an epigenetic mechanism that defines the heterochromatic nature of centromeric DNA [90]. Based on these findings, de novo centromere assembly of CENP-A is known to be dependent on heterochromatin and the RNA interference pathway [100], and once assembled, CENP-A chromatin is propagated by epigenetic means in the absence of heterochromatin [99].

### CENPA a Genomic Target for Drug Development and Human Diseases

It is well accepted that impaired kinetochore assembly and centromere activity are potential causative factors for aneuploidy [101]. Defects on kinetochore organization and function could become lethal to the cells if they suffer substantial damage to their chromosomes. If the damage is low or minimal, the cells may be able to stay alive with accumulation of aneuploid chromosomes which potentially leads to the development of cancer. Molecular participants of the process, such as CENPA, could eventually be exploited as novel therapeutic targets. A mechanism for explaining the generation of aneuploidy could involve mislocalization of CENPA, leading to the genome instability observed during cancer progression. To date, only a few reports have shown a link between CENPA and human diseases. Real-time PCR and tissue microarrays in patients suffering hepatocellular carcinoma (HCC) revealed that the expression level of mRNA encoding CENPA was higher than that in adjacent non-neoplasic liver tissue [102]. The overexpression of CENPA occurs at the transcriptional level and might be related to malignant proliferation of HCC. Similarly, in human colorectal cancer tissues, overexpression of CENPA is believed to play a role in the aneuploidy phenomenon [103]. CENPA was also found overexpressed among various core markers in neoplasic intratubullar germ cells and it served to propose the centromere antigen as a new biological marker of human disease [104]. In *Drosophila*, when the fly homolog of CENPA*,* *CID*, is overexpressed, it was found mislocalized into regions that are normally noncentromeric. CID mislocalization promotes formation of ectopic centromeres and multicentric chromosomes, which causes chromosome missegregation, aneuploidy and growth defects [105].

Currently they are not any drugs specifically acting on CENPA. The shared histone structure of CENPA with other nucleosomal components and the fact that knocking down the function of CENPA causes cells to be non-viable, makes finding a drug specifically targeting CENPA a sizeable challenge. Exposure of human hepatoma cell lines to a low dose of the chemotherapy drug doxorubicin (generic) induces a senescence-like phenotype preceded by multinucleation and down regulation of multiple proteins with mitotic checkpoint functions, including CENPA [106].

As an essential factor for kinetochore assembly, modifying the expression of CENPA and its epigenetic modifications could represent valuable strategies for controlling the process of cell division. Thus, targeting the specific N-terminal domain of CENPA potentially could be used to interfere with the high growth activity of cancer cells. CENPA is specifically phosphorylated at the N-terminal domain in prophase reaching a maximal level in prometaphase. Antibodies generated against a phosphorylation site within CENPA, similar to that around Ser10 of histone H3, served to illustrate a role of kinetochore in cytokinesis [107]. In this context, it should be noted that an antibody generated against the highly charged N-terminal peptide of human CENPA (residues 3–17) microinjected into cultured cells caused arrest in interphase before mitosis and consequently impaired cell division, and led to cell death [108, 109].

On the other hand, the presence of human autoantibodies is a common clinical event in people who have connective tissue diseases or other autoimmune disorders [110]. Autoantibodies against CENPA and CENPB are primarily found in patients suffering limited systemic sclerosis or scleroderma [111]. Human autoimmune patients positive for anticentromere antibodies (ACA+) had significantly more aneuploidy than either ACA-negative patients or non-disease-afflicted healthy controls [112]. The correlation between the presence of ACAs and chromosomal abnormalities suggests that aneuploidy might be the result of non-disjunction secondary to a dysfunction of centromeres [112]. Based in these observations, the variable N-terminal region and the highly conserved C-terminal histone fold domain (HFD) of CENPA are potential targets for drug development, with applications to the fields of chromosome segregation and cell division [11].

## CLOSING REMARKS

Despite the substantial progress made in establish the analysis of the specific DNA sequences at the centromeres in a variety of species and identifying the protein complexes at the kinetochore, much remain to be understood about the biology of this chromosomal region. Recent findings demonstrated that transcription of satellite DNA play a role critical in the establishment of a centromere and are required to promote CENPA over centromeric DNA [99]. It is well established that replacement of histone H3 for CENPA at specific domains of the centromere chromatin is necessary for kinetochore assembly and function. Nucleosomes containing CENPA create a high-order chromatin conformation that generates centromere identity and maintenance of centromere activity is dictated by epigenetic mechanisms. New strategies remain to be applied to achieve the goal of a detailed structural information of CENPA and how the protein interact spatially with other components in the cellular context of a three dimensional model of the kinetochore. In this regards, while this review was in process, recent findings reported the identification of new CENPA binding partners that act as “ring bearers” that bring CENPA to centromeres for assembly [113-117].

Finally, targeting the regulation of expression of CENPA in tumour cells should allow the control of normal kinetochore assembly and proper cell division. The development of drugs that interfere with any cellular regulators of CENPA modification and function is something to consider for interfering with some aspects of tumour cell growth.

## Figures and Tables

**Fig. (1):**
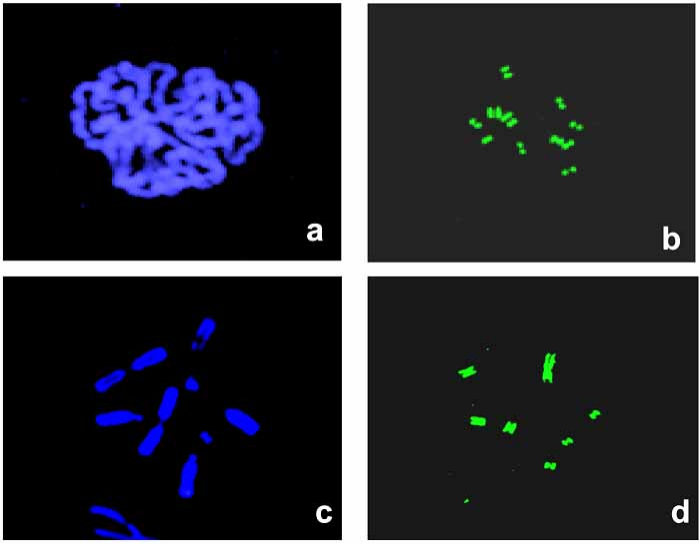
Immunofluorescence staining of centromeres by human autoimmune serum. Human autoantigens including CENPA and CENPB are localized at the inner kinetochore plates of mitotic chromosomes as shown in PtK1 (**b**) and Indian muntjac cells (**d**). DNA staining with DAPI is shown in panels **a**, and **c**.

**Fig. (2):**
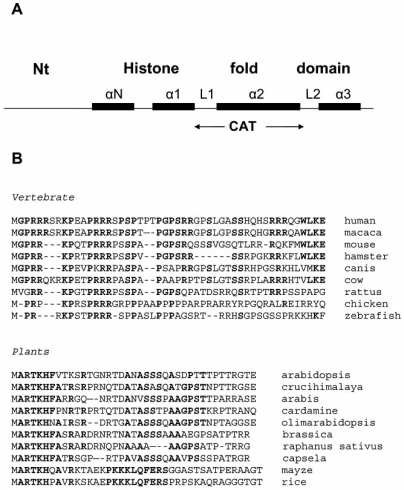
Structural features of centromere protein CENPA. **A**) Organization of the conserved histone fold domain and the variable Nterminal region of human CENPA. All CENPA homologues proteins identified, present the same basic organization at the CATD domain which is the targeting site for centromere localization. Human CENPA share 59% homology in the CATD domain with that of human histone H3. **B**) Comparison of amino acid sequences of the Nt variable region of CENPA in some vertebrates and plants species. In bold are the conserved residues.

**Fig. (3):**
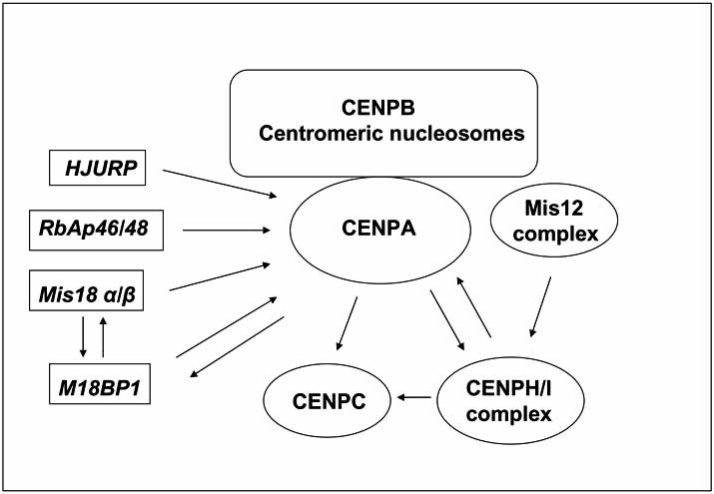
Localization dependencies of human inner kinetochore proteins. The arrows indicate mutual requirements for proper loading at the centromere of kinetochore proteins based on RNAi experiments and immunostaining. At least four different classes of components participate for loading of newly synthesized human CENPA onto the centromeres: the chaperones HJURP and RbAP46/48, the Mis18 complex, and M18BP1. A four subunit hMis12 complex is though to localize to kinetochores in a manner that is at least partially independent of the ENPA pathway in the kinetochore assembly process.
